# A New Ir‐NHC Catalyst for Signal Amplification by Reversible Exchange in D_2_O

**DOI:** 10.1002/chem.201601211

**Published:** 2016-06-03

**Authors:** Peter Spannring, Indrek Reile, Meike Emondts, Philipp P. M. Schleker, Niels K. J. Hermkens, Nick G. J. van der Zwaluw, Bram J. A. van Weerdenburg, Paul Tinnemans, Marco Tessari, Bernhard Blümich, Floris P. J. T. Rutjes, Martin C. Feiters

**Affiliations:** ^1^ Institute for Molecules and MaterialsRadboud UniversityHeyendaalseweg 1356525 AJ NijmegenThe Netherlands; ^2^ RWTH AachenInstitut für Technische und Makromolekulare ChemieWorringerweg 252074 AachenGermany

**Keywords:** biocompatible, carbenes, ligand design, *para*hydrogen, SABRE, water-soluble

## Abstract

NMR signal amplification by reversible exchange (SABRE) has been observed for pyridine, methyl nicotinate, *N*‐methylnicotinamide, and nicotinamide in D_2_O with the new catalyst [Ir(Cl)(IDEG)(COD)] (IDEG=1,3‐bis(3,4,5‐tris(diethyleneglycol)benzyl)imidazole‐2‐ylidene). During the activation and hyperpolarization steps, exclusively D_2_O was used, resulting in the first fully biocompatible SABRE system. Hyperpolarized ^1^H substrate signals were observed at 42.5 MHz upon pressurizing the solution with *para*hydrogen at close to the Earth's magnetic field, at concentrations yielding barely detectable thermal signals. Moreover, 42‐, 26‐, 22‐, and 9‐fold enhancements were observed for nicotinamide, pyridine, methyl nicotinate, and *N*‐methylnicotinamide, respectively, in conventional 300 MHz studies. This research opens up new opportunities in a field in which SABRE has hitherto primarily been conducted in CD_3_OD. This system uses simple hardware, leaves the substrate unaltered, and shows that SABRE is potentially suitable for clinical purposes.

## Introduction

Hyperpolarization (HP) methods enhance nuclear magnetic resonance (NMR) signals, overcoming the fundamental problem of low sensitivity.^1^ Current HP methods include dynamic nuclear polarization (DNP),^2^ *para*hydrogen‐induced polarization (PHIP),^3^, ^4^, ^5^, ^6^ and signal amplification by reversible exchange (SABRE).^7^ DNP^8^, ^9^ and PHIP^10^ have recently led to biomedical applications such as tumor or metabolic imaging in vivo. Ultimately, it is desired to use HP methods in magnetic resonance imaging (MRI) for medical diagnosis.^1^, ^11^, ^12^ For such clinical applications, the methods should meet the following conditions: a) polarization of a wide range of substrates without chemical alteration; b) fast and continuous polarization without contamination; c) heteronuclei with long relaxation times should be polarizable; d) inexpensive hardware and a simple procedure; and e) a biocompatible hyperpolarization medium.^1^ With DNP, nuclear magnetization is amplified by transfer of magnetic hyperpolarization from stable radicals to the nuclei. It can be applied to a variety of substrates without chemical alteration, polarizes heteronuclei, and works in water (conditions a–c and e). On the downside is the required radical and costly setup. PHIP draws nuclear polarization from the transfer of the spin order of *para*hydrogen (*p*‐H_2_) by chemical hydrogenation; it is relatively inexpensive and more rapid than DNP, works efficiently with heteronuclei, and is active in water^6^, ^13^ (conditions b–e), but the substrate is irreversibly modified by hydrogenation. Despite the limitations, both DNP and PHIP have already been used in in vivo MRI applications.^8^, ^9^, ^10^, ^14^, ^15^, ^16^

**Scheme 1:**
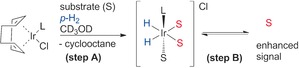
Steps in SABRE. A) Activation of [Ir(Cl)(L)(COD)]‐type catalyst precursor with *p*‐H_2_ and excess substrate (S, red when hyperpolarized) to form [Ir(H)_2_(L)(S)_3_]Cl, B) reversible exchange of S resulting in hyperpolarized S in solution.

SABRE involves nuclear hyperpolarization by synchronous association of substrates and *p*‐H_2_ with a suitable polarization transfer catalyst.^17^ Typical catalyst precursors are square‐planar Ir^I^ complexes bearing bulky electron‐donating ligands (Scheme 1, L), such as *N*‐heterocyclic carbenes (NHCs).^18^, ^19^ Such precursors are activated (Scheme 1, step A) to octahedral Ir^III^ complexes^20^ that contain *cis*‐hydrides and three coordinated substrates, of which the two equatorial ones (S_eq_) exchange rapidly. Hyperpolarization is transferred via the transient scalar coupling network between H and S_eq_,^7^, ^21^ allowing the detection of enhanced NMR signals of dissociated substrate S over several s. A dynamic process with weakly binding S enables large amounts of the substrate to be polarized, resulting in SABRE of non‐coordinated S (Scheme 1, step B).^18^, ^19^, ^22^, ^23^

**Scheme 2:**
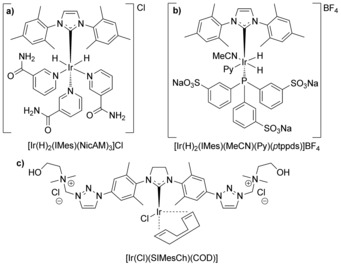
Previous water‐soluble SABRE catalysts with functionality at a) substrate,^27^ b) co‐ligand,^33^ or c) SIMes backbone.^32^, ^33^

[Ir(Cl)(IMes)(COD)]^18^ (IMes=1,3‐bis(2,4,6‐trimethylphenyl)imidazole‐2‐ylidene, COD=1,5‐cyclooctadiene) is the most prominent SABRE catalyst for pyridine (Py) and its derivatives in CD_3_OD.^24^, ^25^, ^26^ Activated [Ir(H)_2_(IMes)(S)_3_]Cl (Scheme 2 a) species are usually generated in situ because of their limited lifetime and susceptibility to side reactions.^25^, ^27^ SABRE rapidly and continuously hyperpolarizes ^1^H nuclei and heteronuclei^28^, ^29^, ^30^, ^31^ in various substrates^24^ with a simple setup (conditions a–d),^19^ but has not yet been accomplished using exclusively pure D_2_O during the activation and hyperpolarization steps.^11^, ^32^ This is due to the apolar IMes ligand, which requires organic solvents for dissolution. Additional complications for SABRE in D_2_O are low H_2_ solubility and rapid proton exchange, which hamper hyperpolarization in systems that otherwise function excellently in organic solvents^33^ or ethanol/water mixtures.^12^ To date, conventional SABRE systems have been rendered water‐soluble in three ways: 1) appending polar groups on the substrate, 2) addition of an axial co‐ligand with polar groups, or 3) incorporation of polar groups at the NHC backbone. The only report concerning the first approach employed organic solvents in step A and has hitherto only been applied to nicotinamide as the substrate (Scheme 2 a).^27^ Efforts concerning the second approach involve addition of water‐soluble phosphines (Scheme 2 b),^34^ yet do not display SABRE because the strongly coordinating ligands interfere with hydrogen and substrate exchange.^33^ The example of the third approach (Scheme 2 c) required organic co‐solvents and showed no hyperpolarization in D_2_O,^32^, ^33^ in line with the report that electronic alterations of IMes and the use of saturated rings (SIMes) have detrimental effects on polarization transfer.^35^ For in vivo SABRE, a straightforward method in D_2_O is needed that avoids the need for organic solvents both in the activation step A and the hyperpolarization step B.

## Results

### Design and synthesis of the water‐soluble SABRE catalyst

**Scheme 3:**
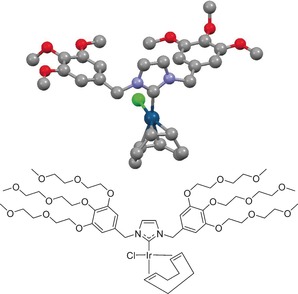
Top: X‐ray structure of [Ir(Cl)(Itome)(COD)] (**1**); bottom: structure of [Ir(Cl)(IDEG)(COD)] (**2**).

We surmise that the apolar nature of the catalysts involved, rather than the low solubility of H_2_ in D_2_O, has hitherto prevented successful SABRE. To date, only little research has been carried out on SABRE using carbenes other than IMes,^35^ in spite of the literature on NHC functionalization with water‐soluble groups.^36^ As functional groups on both aryl moieties have a strong electronic influence^35^ on the carbene due to the conjugation in the ligand, we decided to modify approach 3) above by designing a new Ir‐NHC complex with two benzyl moieties. The methoxy groups in 1,3‐bis(3,4,5‐trimethoxybenzyl)imidazole‐2‐ylidene (Itome) induce electronic effects similar to those of polyethylene glycol derivatives.^37^ Therefore, Itome**⋅**HCl^37^ and 1,3‐bis(3,4,5‐tri(diethyleneglycol)benzyl)‐1*H*‐imidazole iodide (IDEG**⋅**HI) and the corresponding complexes [Ir(Cl)(Itome)(COD)] (**1**) and [Ir(Cl)(IDEG)(COD)] (**2**, Scheme 3) were synthesized. Complex **1** showed SABRE of Py in CD_3_OD (SI), but proved to be insoluble in D_2_O. The X‐ray crystal structure of **1** was elucidated, which showed a square‐planar complex. Of note are the Ir‐C^NHC^ distance of 2.026(3) Å, the Ir‐Cl distance of 2.3737(9) Å, and the Cl‐Ir‐C^NHC^ angle of 89.46(8)°, which are similar to those in [Ir(Cl)(IMes)(COD)] (Ir‐C^NHC^ bond distance 2.055(5) Å, Ir‐Cl distance 2.3527(14) Å, and Cl‐Ir‐C^NHC^ angle 89.82(14)°).^38^ Furthermore, the NHC ligand has a buried volume (%V_bur_) of 27.7 %, a value close to those of conventional SABRE catalysts.^35^ Complex **2** indeed turned out to be soluble in D_2_O and water. The π‐acceptor strength of the ligands was determined by the synthesis of Se (selenourea) analogues of the Itome and IDEG complexes (see the Supplementary Information), which showed similar ^77^Se chemical shifts in CDCl_3_ of *δ*=7.2 and 6.8 ppm (π‐acceptor ability parameter, PAAP^39^, ^40^, ^41^), respectively, lower than that found for the ligand in the best SABRE catalyst in CD_3_OD (*δ*=31.6 ppm^41^), but relatively close to it when the full PAAP range is considered.^40^ Thus, in spite of the replacement of the substituted phenyl moieties in IMes by benzyl moieties in Itome and IDEG, the relevant steric and electronic parameters of the NHC are hardly affected.

### SABRE of complex 1 in CD_3_OD

**Figure 1:**
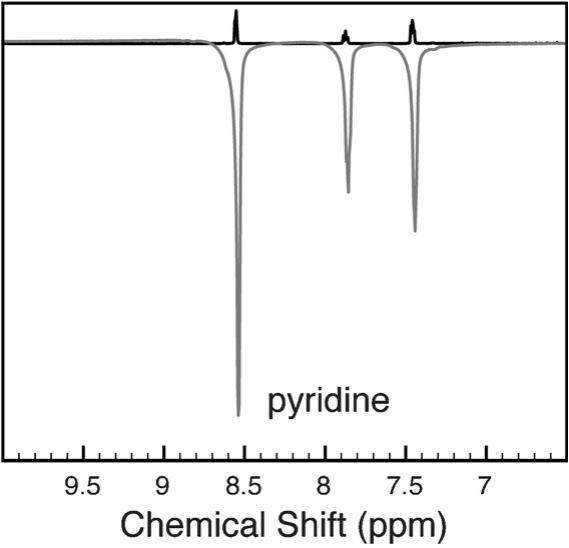
^1^H SABRE experiments on **1** with pyridine in CD_3_OD on a 600 MHz spectrometer (aromatic region, single scans). Black trace: thermal polarization spectrum. Grey trace: hyperpolarized spectrum.

The investigation of SABRE hyperpolarization with these complexes commenced with conventional 600 MHz experiments on complex **1** in CD_3_OD in a Wilmad “thin‐wall” pressure tube at 65 G polarization transfer field (PTF), as previously performed by our group.^19^ Experiments at ambient temperature using complex **1** (1 mm in CD_3_OD) and 10 mm of pyridine with 5 bar of 51 % *p*‐H_2_ showed hyperpolarization of all signals of free Py (**S1**, Figure 1), which was maximal after 48 h of activation (time after the sample was pressurized with 5 bar *p*‐H_2_, slowly forming [Ir(H)_2_(Itome)(Py)_3_]Cl with one hydride signal at *δ*=−22.17 ppm (see the Supplementary Information). The enhancements at 600 MHz were 15‐, 11‐, and 7‐fold for the *o‐*, *p‐*, and *m‐* ^1^H signals at *δ*≈8.6, 7.8, and 7.4 ppm in free pyridine, respectively. These enhancement factors are typically 6‐ to 7‐fold lower than those in experiments with the conventional catalyst [Ir(Cl)(IMes)(COD)] performed under similar conditions.^19^ Moreover, the hydride signal in the activated complex was enhanced 8‐fold (see the Supplementary Information). The enhanced signals indicate that this type of Ir‐NHC complex is indeed suitable for SABRE‐type hyperpolarizations. The results acquired with **1** in CD_3_OD could in theory be extrapolated to hyperpolarizations of **2** in D_2_O, given the electronic similarity of the complexes.

### SABRE of complex 2 in D_2_O at 42.5 MHz

**Figure 2:**
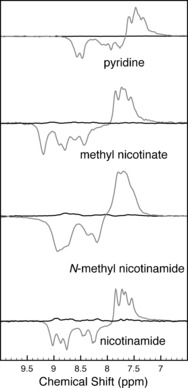
^1^H SABRE experiments on **2** in D_2_O on a 42.5 MHz spectrometer (aromatic region, single scans). Top to bottom: **S1**, **S2**, **S3**, **S4**. Black traces: thermal polarization spectra, grey traces: hyperpolarized spectra.

Initial hyperpolarization experiments in D_2_O were performed on a 42.5 MHz NMR benchtop spectrometer (conditions: 48 h activation, 1 mm **2**, 10 mm Py (**S1**), Wilmad “middle‐wall” pressure tube, 6 bar of 92 % *p*‐H_2_, 30 s heating at 50–55 °C, and shaking for 5 s at close to the Earth's magnetic field (1 G around the spectrometer)). This resulted in immediate and strong hyperpolarization of free Py compared to the thermally polarized signals at room temperature (Figure 2). The substrate scope was extended to the *m*‐substituted derivatives methyl nicotinate **S2**, *N*‐methylnicotinamide **S3**, and nicotinamide **S4** (conditions: 24 h activation, 0.66 mm **2** and 13.32 mm substrate, “thin‐wall” pressure tube, 0.6 mL sample, 5.5 bar), for which the thermally polarized aromatic signals were barely detectable under these circumstances. The signal enhancements were estimated to be in the 50‐ to 200‐fold range. The results with all of the substrates indicated that the current method enables substrate hyperpolarization in D_2_O through a rapid, straightforward procedure that can be carried out at close to the Earth's magnetic field. This system also shows that a relatively inexpensive benchtop spectrometer is sufficient for detecting the hyperpolarized analytes of interest. Such devices are generally compact and can have diverse applications, such as in quality control and drug detection,^42^ as many drugs contain *N*‐heterocyclic six‐membered aromatic moieties.

### SABRE of complex 2 in D_2_O at 300 MHz

**Figure 3:**
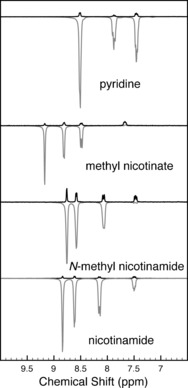
Single‐scan ^1^H SABRE experiments using **2** in D_2_O on a 300 MHz spectrometer (aromatic region). Top to bottom: **S1**, **S2**, **S3**, **S4**. Black traces: thermal polarization spectra; grey traces: hyperpolarized spectra.

Measurements at higher magnetic field (300 MHz) involved polarization transfer in the stray field of the superconducting magnet (conditions: 1 mm **2** in D_2_O, Wilmad “thin‐wall” pressure tube, 10 or 20 equiv. of **S1**–**S4**, 30 s heating at 60 °C, 5 s polarization transfer at 80 G, Figure 3). The activated complex was slowly formed, with the hydride signal at *δ*=−22.17 ppm (see the Supplementary Information). The sample cooled to approximately 40–45 °C during the polarization transfer. **S1**–**S3** gave good enhancement factors with reference to the thermal signals of 24, 14, and 26, respectively (Table 1, entries 1–3). For **S4**, all four aromatic signals were polarized, yielding enhancements up to 42‐fold (entry 4). The nicotinamide hyperpolarization of **S4** gave signal enhancements comparable to those found for [Ir(Cl)(IMes)(COD)] following dissolution in an organic solvent, dilution with D_2_O, and subsequent evaporation of the solvent,^27^ provided that the difference in magnetic field (7.0 vs. 9.4 T) is taken into account, albeit for a lower concentration of **S4** (10 vs. 35 mm). The system presented here requires only manual shaking, no additional specialized hardware for *p*‐H_2_ bubbling,^27^ and can be applied to several substrates.

**Table 1:** Signal enhancement values (ɛ) of **S1–S4** with **2** in D_2_O.^[a]^

Entry	S	**2** (mol %)	Activation	ɛ(H_a_)	ɛ(H_b_)	ɛ(H_c_)	ɛ(H_d_)
1	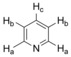	10 %	48 h	24	14	26	–
2^[b]^	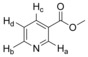	5 %	18 h	22	10	9	0
3^[b]^	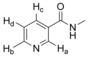	5 %	18 h	9	7	6	0
4	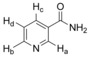	10 %	18 h	42	28	30	8

[a] ^1^H SABRE experiments at 80 G PTF, 1 mm **2**, 10 mm **S1–S4** in D_2_O, 5.5 bar of 92 % *p*‐H_2_, 48 h activation, 60 °C heating for 30 s, 5 s shaking, detection at 300 MHz, ɛ for free substrate. [b] 0.66 mm **2**, 13.32 mm in D_2_O, 18 h activation. Protons are labeled a, b, etc. in order of descending δ.

## Discussion

The SABRE method reported here with catalyst precursor [Ir(Cl)(IDEG)(COD)] (**2**) is the first that operates in D_2_O without organic solvents in the catalyst activation and substrate hyperpolarization steps. In previous systems, water solubility was achieved either by the use of a very polar substrate,^27^ a water‐soluble co‐ligand,^33^ or appending a polar group on an SIMes backbone,^33^ and activation in organic solvents was required. Here, water solubility has been achieved by polar group incorporation through the design of a new NHC ligand containing diethyleneglycol chains on the benzyl substituents at the imidazolium ring. The activation and hyperpolarization processes could be accomplished in neat D_2_O. Nicotinamide is an interesting biological substrate in the sense that it can potentially be used to probe metabolic processes in vivo.^31^ The obtained 42‐fold enhancement with nicotinamide is approximately equivalent to the hyperpolarization found with Ir‐IMes derivatives in [D_6_]EtOH/D_2_O with this substrate.^27^ Furthermore, we have shown here that hyperpolarization of similar magnitude can also be obtained in D_2_O with less polar substrates, such as the methyl ester or methyl amide analogues, or Py. An additional advantage is that continuous *p*‐H_2_ bubbling^27^ through the whole sample is not required; rather, merely shaking the closed vessel is sufficient, and hyperpolarizations can be achieved at close to the Earth's magnetic field on a benchtop spectrometer.

## Conclusion

Based on a new Ir‐NHC catalyst, [Ir(Cl)(IDEG)(COD)], we report here the first SABRE system to operate exclusively in D_2_O, that is, without the need for an organic solvent in the activation or other steps. The substrate scope demonstrated herein, ranging from pyridine, methyl nicotinate, and *N*‐methylnicotinamide to nicotinamide, suggests that more biologically important substrates should be hyperpolarizable in this way, as many pharmaceutical drugs contain heterocyclic aromatics.^43^, ^44^ This work clearly proves that the low solubility of H_2_ does not preclude the possibility of SABRE in pure water.^27^ We envisage that these results will inspire research on new SABRE applications in water, significantly extending the opportunities that have hitherto been limited to the use of SABRE in CD_3_OD. The hyperpolarizations performed in D_2_O have been achieved by straightforward methods and on relatively inexpensive instruments. This newly developed water‐soluble, organic solvent‐free SABRE system is the first and most important step towards biomedical in vivo applications.

## Experimental Section

**Synthesis of [Ir(Cl)(Itome)(COD)] (1)**: Itome**⋅**HCl^37^ (1 equiv, 857 mg, 1.84 mmol) was mixed with Ag_2_O (0.5 equiv, 214 mg, 0.92 mmol) in dry 1,2‐dichloroethane (30 mL) under inert conditions in the dark, and the mixture was heated under reflux for 24 h. Subsequently, the mixture was cooled, [Ir(Cl)(COD)]_2_ (0.5 equiv, 619 mg, 0.92 mmol) was added, and the resulting mixture was heated under reflux for a further 24 h. After cooling to room temperature, it was filtered through Celite, the solvent was evaporated, and the compound was additionally purified by flash column chromatography (neutral Al_2_O_3_; CHCl_3_/MeOH, 99:1), collecting the first fraction. Yield: 82 %, yellow powder (1.154 g, 1.51 mmol). Crystals suitable for X‐ray crystallography were obtained by diffusion of diethyl ether into a saturated solution of **1** in CH_2_Cl_2_. ^1^H NMR (CDCl_3_, 500 MHz): *δ*=6.74 (s, 4 H; C(OMe)C*H*C_q_), 6.73 (s, 2 H; NC*H*=C), 6.14 (d, 2 H, ^3^ *J*=14 Hz; CH_2_C*H*=CH), 5.17 (d, 2 H, ^3^ *J*=14 Hz; CH_2_C*H*=CH), 5.14 (s, 2 H), 4.68 (br s, 2 H; benzylic C_q_C*H*HN), 3.86 (m, 12 H; *m*‐OC*H*_3_), 3.84 (m, 6 H; *p*‐OC*H*_3_), 3.02 (br s, 2 H; benzylic C_q_C*H*HN), 2.23 (s, 2 H), 1.8–1.7 (m), 1.7–1.6 ppm (m); ^13^C NMR (CDCl_3_, 500 MHz): *δ*=180.5, 153.3, 137.9, 131.9, 120.5, 105.7, 85.2, 60.8, 56.4, 54.5, 51.9, 33.6, 29.6 ppm; elemental analysis calcd (%): C 48.71, H 5.27, N 3.67; found: C 48.81, H 5.27, N 3.52.

**Synthesis of [Ir(Cl)(IDEG)(COD)] (2)**: The procedure was similar to that used for compound **1**, but using IDEG**⋅**HI (see the Supplementary Information). Yield of **2**: 78 %, yellow/brown oil (666 mg, 0.51 mmol). ^1^H NMR (CDCl_3_, 500 MHz): *δ*=6.69 (s, 4 H; C(OMe)C*H*C_q_), 6.68 (s, 2 H; NC*H*=C), 5.87 (d, 2 H, ^3^ *J*=15 Hz; CH_2_C*H*=CH), 5.27 (d, 2 H, ^3^ *J*=15 Hz; CH_2_C*H*=CH), 4.64 (s, 2 H; benzylic C_q_C*H*HN), 4.16 (m), 3.85 (t), 3.81 (t), 3.74–3.70 (m), 3.57–3.55 (m), 3.39 (m, 6 H; *p*‐OC*H*_3_), 3.38 (m, 12 H; *m*‐OC*H*_3_), 2.97 (br s, 2 H; benzylic C_q_C*H*HN), 2.0–1.6 (m), 1.3–0.9 ppm (m); ^13^C NMR (CDCl_3_, 500 MHz): *δ*=180.4, 152.9, 138.2, 131.7, 120.5, 107.9, 85.0, 72.4, 72.0, 71.9, 70.6, 70.5, 70.4, 69.7, 69.0, 59.0, 54.3, 52.0, 33.6, 29.5 ppm; elemental analysis calcd (%): C 51.09, H 6.86, N 2.17; found C 51.11, H 7.06, N 2.07.

## Supporting information

As a service to our authors and readers, this journal provides supporting information supplied by the authors. Such materials are peer reviewed and may be re‐organized for online delivery, but are not copy‐edited or typeset. Technical support issues arising from supporting information (other than missing files) should be addressed to the authors.

Supplementary
