# Supplementary material for: A New Ir‐NHC Catalyst for Signal Amplification by Reversible Exchange in D2O
Source: Chemistry. 2016 Jun 3;22(27):9277–82. doi: 10.1002/chem.201601211 (PMC5089654; doi:10.1002/chem.201601211)
Supplement: Supplementary file 1 — Supplementary [file CHEM-22-9277-s001.pdf]

CHEMISTRY
A European Journal

Supporting Information

A New Ir-NHC Catalyst for Signal Amplification by Reversible Exchange in D_2O

Peter Spannring,[a] Indrek Reile,[a] Meike Emondts,[b] Philipp P. M. Schleker,[b] Niels K. J. Hermkens,[a] Nick G. J. van der Zwaluw,[a] Bram J. A. van Weerdenburg,[a] Paul Tinnemans,[a] Marco Tessari,[a] Bernhard Blümich,[b] Floris P. J. T. Rutjes,*[a] and Martin C. Feiters*[a]

chem_201601211_sm_miscellaneous_information.pdf

Supporting Information pertaining to Chem Eur J manuscript chem.201601211

A new Ir-NHC-catalyst for signal amplification by reversible exchange in D_2O

Peter Spannring,[a] Indrek Reile,[a] Meike Emondts,[b] Philipp P. M. Schleker,[b] Niels K. J. Hermkens,[a] Nick G. J. van der Zwaluw,[a] Bram J. A. van Weerdenburg,[a] Paul Tinnemans,[a] Marco Tessari,[a] Bernhard Blümich,[b] Floris P. J. T. Rutjes[a*] and Martin C. Feiters[a*]

a Radboud University Nijmegen, Institute for Molecules and Materials, Heyendaalseweg 135, 6525 AJ Nijmegen, the Netherlands
b RWTH Aachen, Institut für Technische und Makromolekulare Chemie, Worringerweg 2, 52074 Aachen, Germany

Table of Contents

General procedures 1
Materials 1
Instrumentation and hyperpolarization procedures 1
X-Ray structure determination 2
Ligand and complex synthesis 3
Synthesis of selenoureas 7
SABRE of complex 1 in CD_3OD at 600 MHz 8
SABRE of complex 2 in D_2O at 300 MHz 9
References 12

General procedures

Materials

1,2-Dichloroethane was obtained as dry solvent from Sigma Aldrich at 99% purity. Ag_2O was obtained from Sigma Aldrich at 99% purity. $[Ir(Cl)(COD)]_2$ (99% purity) was purchased at Strem Chemicals. D_2O and CD_3OD (Sigma Aldrich, 99.9%) were used as is, oxygen was removed by freeze-pump-thaw techniques in triplo. $CDCl_3$ was obtained from Cambridge Laboratory Isotopes, as 99.9% D grade, 0.05% V/V TMS. KI was purchased at Merck with 99.5% purity grade. CsF was acquired from Sigma Aldrich with 99% purity. Imidazole was obtained from Acros Organics, with 99% purity. Dry acetonitrile (MeCN, 99.9%) and tetrahydrofuran (THF, not stabilized, 99.8%) were purchased from Actual Chemicals, connected to the solvent purification system MB SPS-800. 1.0 M KOtBu in THF was obtained from Sigma Aldrich. $CHCl_3$ and MeOH were used as technical grade solvents.

Instrumentation and hyperpolarization procedures

Conventional NMR spectra were recorded at 298 K on a Bruker Avance II 500 MHz spectrometer equipped with a BBI probe in Nijmegen, The Netherlands. SABRE with complex **1** was performed on a Bruker Avance III 600 MHz spectrometer equipped with a cryo-cooled HCN probe in Nijmegen using 5 bar of 51% parahydrogen. SABRE with complex **2** was performed at the RWTH in Aachen, Germany, on a 42.5 MHz Magritek Spinsolve spectrometer and a Bruker Avance II 300 MHz spectrometer equipped with a BBO 300 MHz S1 5 mm probe. Measurements at 42.5 MHz involved samples in a middle wall Wilmad LabGlass 9-inch pressure valve NMR tube with 0.2 mL sample volume for

pyridine, pressurized with 6 bar parahydrogen with 92% *para* isomer enrichment. For substrates methyl nicotinate, *N*-methyl nicotinamide and nicotinamide, a thin wall Wilmad LabGlass 9-inch NMR tube was used with 0.7 mL sample volume and 5.5 bar 92% parahydrogen. The hyperpolarization was achieved by manually shaking the sample for 5 seconds at a magnetic field of approximately 1 G and rapidly inserting into the bore. At 300 MHz, samples were also loaded with 0.7 mL sample volume, 92% parahydrogen at a pressure of 5.5 bar. The 92% parahydrogen was obtained from a Bruker parahydrogen generator, with a conversion temperature of 37 K. The reported chemical shifts (in ppm) are referenced to the residual solvent signals of CD_3OD and D_2O. NMR samples in high-field SABRE experiments were prepared using volumes of 0.7 mL in a thin wall 5 mm Wilmad quick pressure valve NMR tube of 7-inch length (See Figure S1). Parahydrogen used in SABRE experiments at 500 and 600 MHz was enriched to 51% *para* isomer following the procedure:[1] 4 bar of commercially available dihydrogen gas was stored for 2 hours over activated charcoal at 77 K using liquid nitrogen. The conversion of *o*-H_2 into *p*-H_2 is catalyzed by activated charcoal, which is otherwise symmetry forbidden. Prior to pressurization, the samples were quickly evacuated, before 5 or 5.5 bar was introduced over 15 seconds. The hyperpolarization was achieved by manually shaking the sample for either 5 or 20 seconds at a magnetic field of 65 G or of approximately 80 G and rapidly inserting into the bore. After insertion of the sample, a 90° rf pulse was applied immediately. Due to T1 relaxation, the observed polarization is optimal when the sample is entered smoothly and without time delay into the detector. Enhancement factors were obtained by dividing the absolute substrate integral in the hyperpolarized state by the absolute integral at the thermally polarized state. Both thermal and hyperpolarized spectra were recorded at identical experimental parameters, the thermally polarized signal was recorded after a three minute relaxation delay to ensure full relaxation to thermal equilibrium. High-resolution mass spectra were recorded on a JEOL AccuTOF (ESI). Elemental microanalyses were carried out by the Mikroanalytisches Laboratorium KOLBE, Mülheim an der Ruhr, Germany.

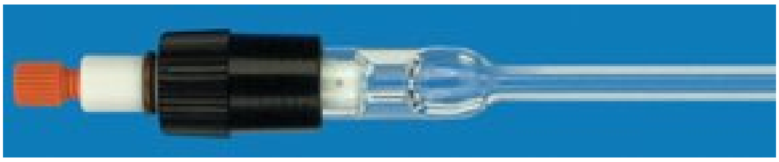
Figure S 1.Picture of a 7-inch Wilmad quick pressure valve NMR tube (www.sigmaaldrich.com)

X-Ray structure determination

For single-crystal X-ray diffraction, a single-crystal was cut to size and mounted on a Mitagen Microloop using high viscosity oil and shock frozen to 208K using liquid nitrogen. Intensity data were collected at 208K. The measurement was performed was performed on a Nonius KappaCCD single-crystal diffractometer (φ and ω scan mode) using graphite monochromated Mo Kα radiation. Diffraction images were integrated using Eval14.[2] Intensity data were corrected for Lorentz and polarization effects. A semiempirical multiscan absorption correction was applied (SADABS).[3] The structure was solved using SHELXT.[4] Refinement was performed with standard methods:refinement against F2 of all reflections with SHELXL-2014. All nonhydrogen atoms were refined with anisotropic temperature factors. The positions of the hydrogen atoms could initially be determined using a difference Fourier map. Hydrogens were subsequently, when possible, replaced by hydrogens at calculated positions and refined riding on the parent atoms.

Ligand and complex synthesis

The Itome ligand was synthesized as imidazolium.HCl salt, published elsewhere,[5] the Itome carbene was formed in-situ by the addition of Ag_2O. 3,4,5-tris-(2-(2-methoxyethoxy)ethoxy)benzyl chloride was synthesized according to a previously published method.[6] The IDEG ligand was synthesized as imidazolium .HI salt, the carbene was formed in a similar way is with Itome.

Synthesis of [Ir(Cl)(Itome)(COD)] (**1**).

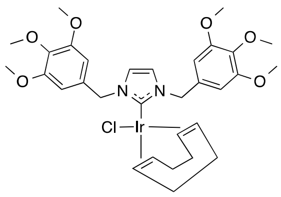

Itome.HCl ligand (1 equiv., 857 mg, 1.84 mmol) was mixed with Ag_2O (0.5 equiv., 214 mg, 0.92 mmol) in 30 mL dry 1,2-dichloroethane under inert conditions in the dark and refluxed for 24 h. Subsequently, the mixture was cooled, and $[Ir(Cl)(COD)]_2$ (0.5 equiv., 619 mg, 0.92 mmol) was added and refluxed for additional 24 h. After cooling to room temperature, it was filtered over celite, the solvent was evaporated and the compound was additionally purified over flash column chromatography (neutral Al_2O_3, $CHCl_3$:MeOH 1%), collecting the first fraction. Yield: 82% yellow powder (1.154 g, 1.51 mmol). Crystals suitable for X-Ray crystallography were obtained by diffusion of diethyl ether into a saturated solution of **1** in DCM. ^{1}H NMR ($CDCl_3$, 500MHz): 6,74 (s, 4H, C(OMe)C*H*C$_q$); 6,73 (s, 2H, NC*H*=C); 6.14 (d, 2H, ^{3}J = 14 Hz, CH_2C*H*=CH); 5.17 (d, 2H, ^{3}J = 14 Hz, CH_2C*H*=CH); 5.14 (s, 2H); 4.68 (broad s, 2H, benzylic C$_q$C*H*HN); 3.86, (m, 12H, *m*-OC*H*$_3$); 3.84 (m, 6H, *p*-OC*H*$_3$); 3.02 (broad s, 2H, benzylic C$_q$C*H*HN); 2.23 (s, 2H); 1.8-1.7 (m); 1.7-1.6 (m). ^{13}C NMR ($CDCl_3$, 500MHz): 180.5; 153.3; 137.9; 131.9; 120.5; 105.7; 85.2; 60.8; 56.4; 54.5; 51.9; 33.6; 29.6. Elemental analysis: C, found 48.81, calculated 48.71; H, found 5.27, calculated 5.27; N, found 3.52, calculated 3.67.

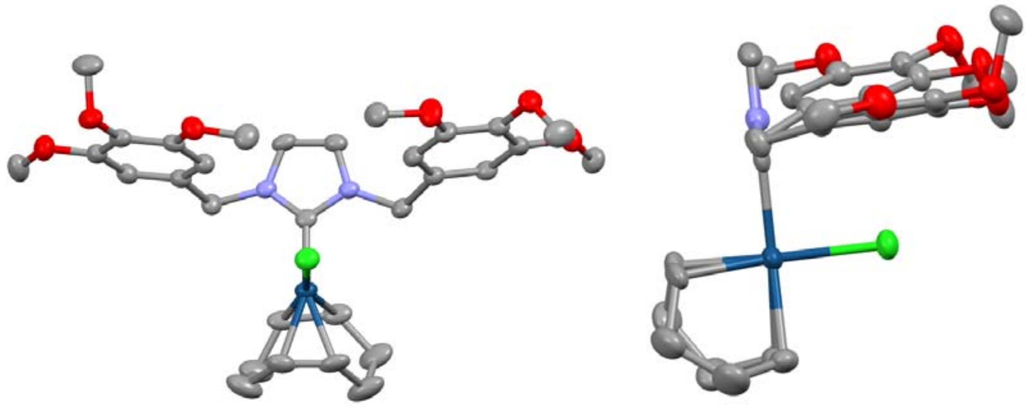

Figure S 2. 50% probability ellipsoids of the X-Ray structure of **1**. Left: front view, right: side view. Atom labeling: Ir: (dark blue); Cl: (green); N: (purple); O (red); C: (grey). H atoms were omitted for clarity.

The X-ray structure of **1** was elucidated, showing a square-planar complex, an Ir-carbene distance of 2.026(3) Å and the NHC ligand has a buried volume (%V_{bur}) of 27.7%, a value close to the conventional SABRE-catalysts.[7] Full characterization of the X-ray is given in the .cif file. The structure has been deposited at the Cambridge Crystallographic Data Centre with deposition number CCDC 1442685.

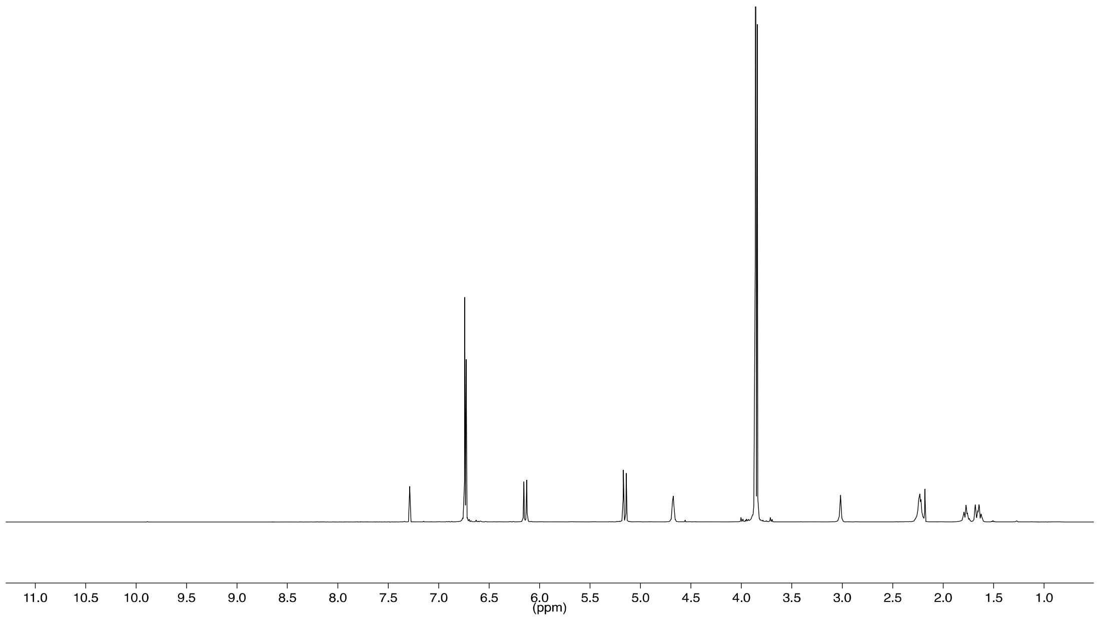

Figure S 3. ^{1}H NMR spectrum of [Ir(Cl)(Itome)(COD)] (**1**) in $CDCl_3$, measured at 500 MHz.

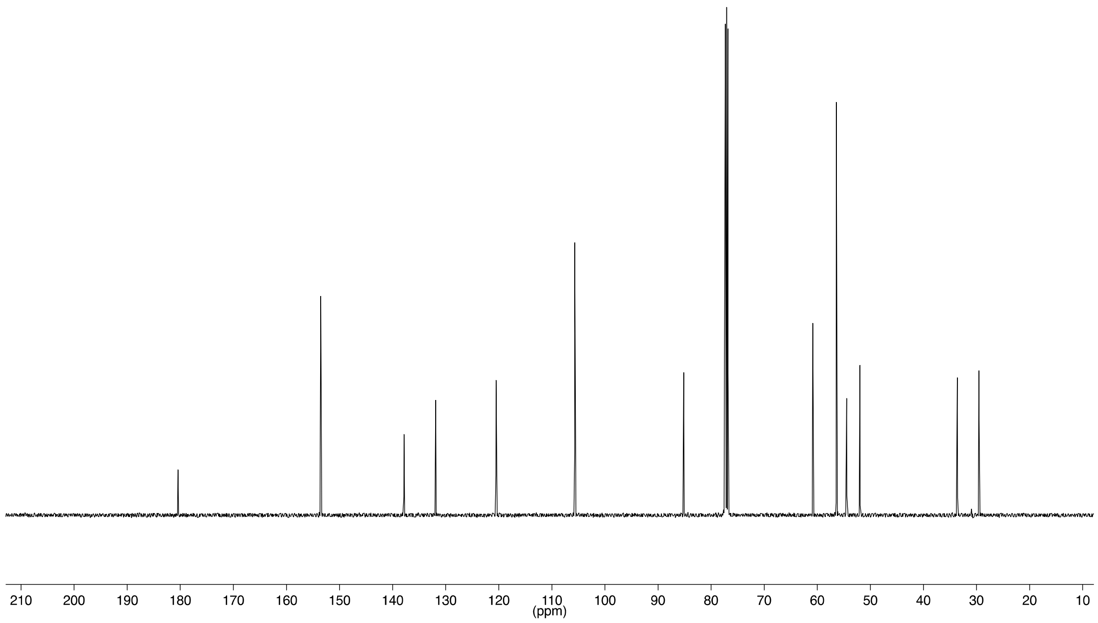

Figure S 4. ^{13}C NMR spectrum of [Ir(Cl)(Itome)(COD)] (**1**) in $CDCl_3$, measured at 500 MHz.

The observed enhancements for the *ortho*, *para* and *meta* signals of free pyridine under the observed conditions were 15, 11 and 7-fold. Under similar conditions enhancements using [Ir(Cl)(IMes)(COD)] after 1 h of activation were around 60-70-fold. The latter catalyst generally decomposes after 48 h activation periods.

Synthesis of IDEG.HI

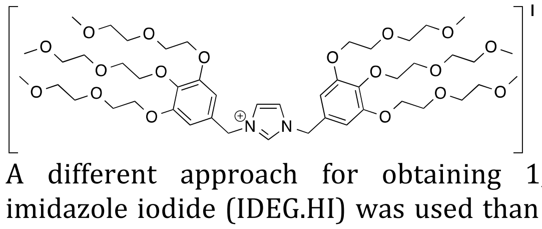

A different approach for obtaining 1,3-bis(3,4,5-tris(diethyleneglycol)benzyl)-1H-imidazole iodide (IDEG.HI) was used than previously reported for the synthesis of 1,3-

bis(3,4,5-tris(tetraethyleneglycol)benzyl)-1H-imidazole chloride,[5] as using CsF-Celite in our case rendered only marginal amounts of product, yielding predominantly monosubstituted product. Instead, the following procedure was applied: 3,4,5-tris(2-(2-methoxyethoxy)ethoxy)benzyl chloride (911 mg, 1.90 mmol, 2 equiv.),[6] was mixed with imidazole (64.6 mg, 0.95 mmol, 1 equiv.), CsF (1.44 g, 9.5 mmol, 10 equiv.) and KI (1.57 g, 10 equiv.) in dry MeCN (20 mL) and refluxed for 24 h. After cooling it was filtered over celite, evaporated to dryness and further purified by flash column chromatography (neutral Al_2O_3, $CHCl_3$:MeOH 1%, gradient to 3%). The first fraction was obtained, after performing the purification in triplo, the combined fractions formed a yellow oil (657 mg, 0.61 mmol, 64%). ^{1}H NMR ($CDCl_3$, 500MHz): 10.52 (s, 1H, NC*H*N); 7.11 (s, 2H, NC*H*=C); 6.82 (s, 4H, C_qCHC_q); 5.27 (s, 4H, benzylic H); 4.24 (t, 8H, ^3J = 9 Hz); 4.20 (t, 4H, ^3J = 10 Hz); 3.87 (t, 8H, ^3J = 9 Hz); 3.82 (t, 6H, ^3J = 9 Hz); 3.74-3.72 (m); 3.58-3.56 (m); 3.40-3.39 (m). ^{13}C NMR ($CDCl_3$, 500MHz): 153.4; 139.5; 136.8; 127.3; 121.3; 109.1; 77.6; 72.4; 72.0; 71.9; 70.6; 70.5; 70.4; 69.7; 69.4; 59.0; 53.9. ESI-MS: IDEG+H: calculated: 957.5171, found: 957.5142. Elemental analysis: found: C, 51.60; H, 8.07; N, 2.23; IDEG.HI: calculated: C, 52.03, H, 7.15; N, 2.58.

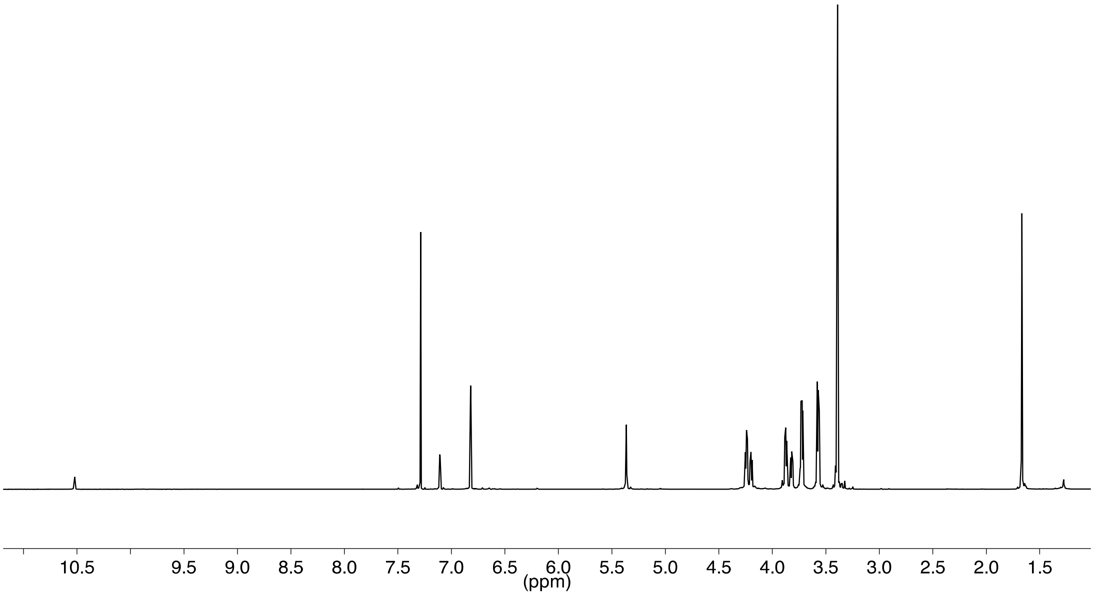

Figure S 5. ^{1}H NMR spectrum of IDEG.HI, in $CDCl_3$ recorded at 500 MHz.

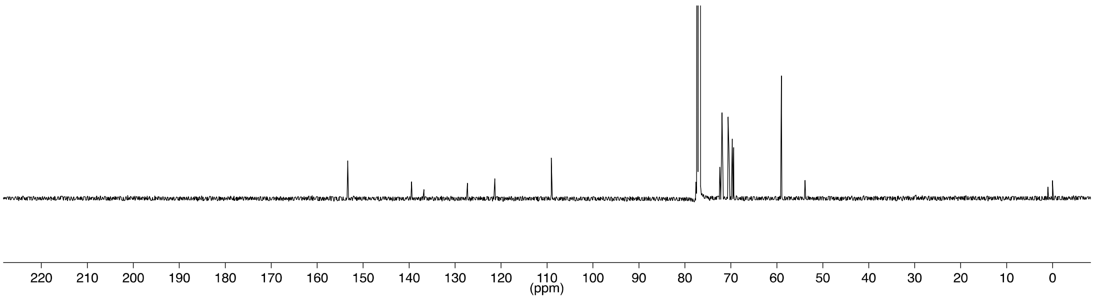

Figure S 6. ^{13}C NMR spectrum in $CDCl_3$ of IDEG.HI recorded at 500 MHz.

Synthesis of [Ir(Cl)(IDEG)(COD)] (**2**).

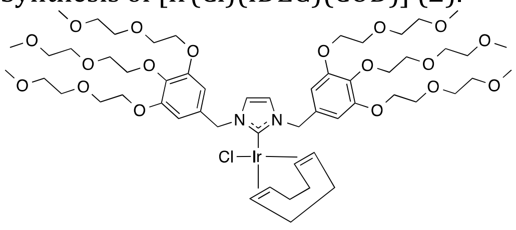

A similar procedure was performed as with compound **1**, using IDEG.HI. Yield of **2**: 78% yellow/brown oil (666 mg, 0.51 mmol). ^{1}H NMR ($CDCl_3$, 500MHz): 6.69 (s, 4H, C(OMe)C*H*C_q); 6.68 (s, 2H, NC*H*=C); 5.87 (d, 2H, ^{3}J = 15 Hz, CH_2C*H*=CH); 5.27 (d, 2H, ^{3}J = 15 Hz, CH_2C*H*=CH); 4.64 (s, 2H, benzylic C_qC*H*HN); 4.16 (m); 3.85 (t); 3.81 (t); 3.74-3.70 (m); 3.57-3.55 (m); 3.39 (m, 6H, *p*-OCH_3); 3.38 (m, 12H, *m*-OCH_3); 2.97 (broad s, 2H, benzylic C_qC*H*HN); 2.0-1.6 (m); 1.3-0.9 (m). ^{13}C NMR ($CDCl_3$, 500MHz): 180.4; 152.9; 138.2; 131.7; 120.5; 107.9; 85.0; 72.4; 72.0; 71.9; 70.6; 70.5; 70.4; 69.7; 69.0; 59.0; 54.3; 52.0; 33.6; 29.5. Elemental analysis: C, found 51.11, calculated 51.09; H, found 7.06, calculated 6.86; N, found 2.07, calculated 2.17.

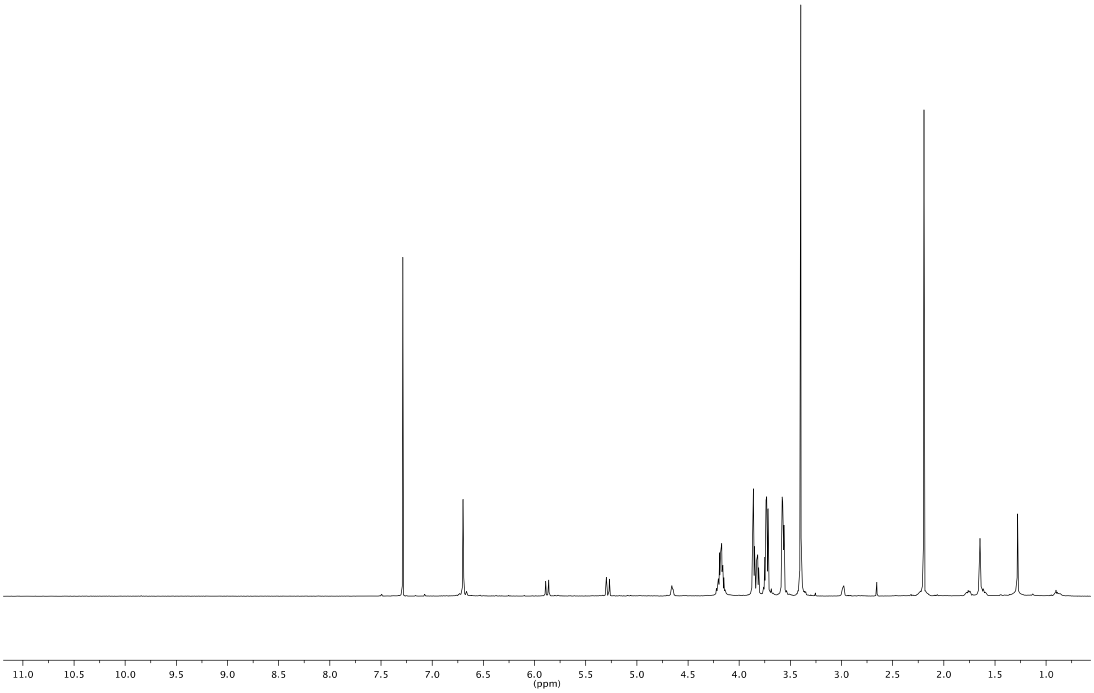

Figure S 7. ^{1}H NMR spectrum of [Ir(Cl)(IDEG)(COD)] (**2**) in $CDCl_3$, measured at 500 MHz.

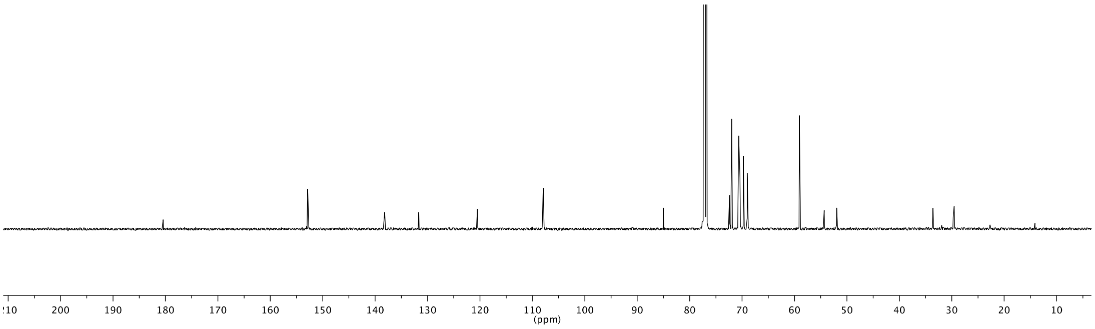

Figure S 8. ^{13}C NMR spectrum of [Ir(Cl)(IDEG)(COD)] (**2**) in $CDCl_3$, measured at 500 MHz.

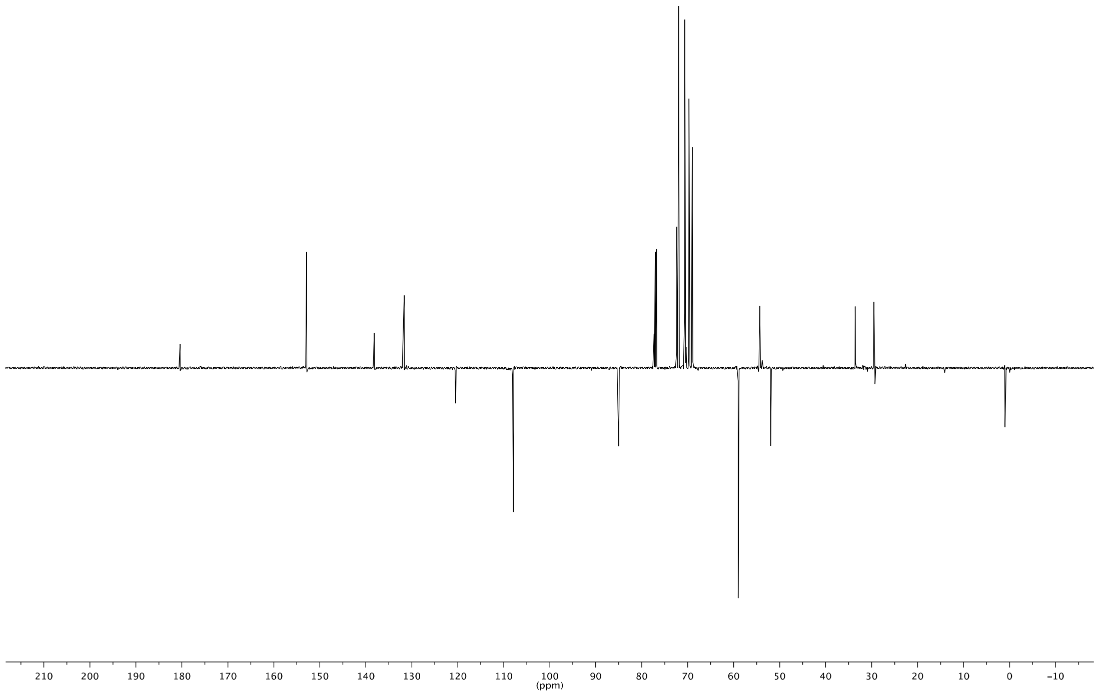

Figure S 9. APT NMR spectrum of [Ir(Cl)(IDEG)(COD)] (**2**) in $CDCl_3$, measured at 500 MHz.

Synthesis of selenoureas

The π-acceptor strength of the ligands was determined by the synthesis of Se (selenourea) analogues of the Itome and IDEG complexes. The selenoureas were synthesized according to a previously published method. (ref: DOI:10.1039/C4SC03264K.)

Safety note: selenium and organoselenium compounds are highly toxic, and should be handled with care. The NHC salt (ca. 50 mg, weighed accurately), excess selenium (ca. 30 mg, pellets, grinded before use) and a stirring bar were added to a Schlenk tube and purged with nitrogen. Next, dry degassed THF (0.75 mL) and KOtBu (1.2 equiv., 1.0 M solution in THF) were added via the septum and the resulting suspension was stirred at room temperature overnight. The solvent was evaporated and the resulting residue was suspended in DCM (ca. 2 mL) and filtered through a pad of celite. The pad was washed with further DCM (ca. 2 mL). The DCM was evaporated and the residue was washed with pentane (3 x ca. 1 mL). The ^{77}Se chemical shift scales were calibrated to the ^{1}H spectrum using the unified Ξ scale according to Harris *et al.*[8]

Synthesis of Se(Itome)

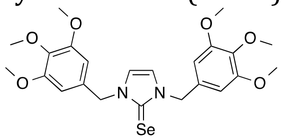

According to the above described method, Se(Itome) was obtained as an brownish solid in 80% yield (49.0 mg).^{1}H NMR ($CDCl_3$, 400MHz): 6.67 (s, 4H), 6.63 (s. 2H), 5.30 (s, 4H), 3.84 (m, 12H), 3.81 (m, 6H); ^{13}C NMR ($CDCl_3$, 100 MHz): 153.60, 138.09, 131.09, 118.78, 105.78, 60.89, 56.31, 53.65; ^{77}Se NMR (76 MHz, $CDCl_3$) δ 7.2 (s); HRMS (ESI^+) calcd for $C_{23}H_{28}N_2O_6Se$ $[M+H]^+$ 509.1191, found 509.1193.

Synthesis of Se(IDEG)

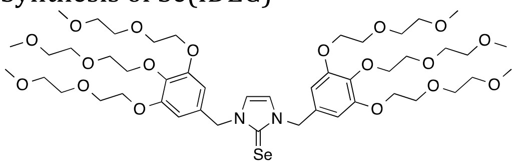

According to the above described procedure, 14 mg of IDEG.HI was converted into the desired selenourea and was obtained as an brownish/red solid in 67 % yield (9 mg) ^{1}H NMR ($CDCl_3$, 500MHz): 6.69 (s, 2H); 6.64 (s, 4H); 5.23 (s, 4H); 4.16-4.13 (m, 12H), 3.85-3.83 (m, 8H), 3.80-3.78 (m, 4H), 3.72-3.69 (m, 12H), 3.56-3.54 (m, 12H), 3.38 (s, 6H), 3.37 (s, 12H); ^{13}C NMR ($CDCl_3$, 500MHz): 152.98, 138.46, 130.93, 118.82, 108.48, 72.50, 72.09, 70.81, 70.65, 70.52, 69.82, 69.07, 59.21, 59.18, 53.60; ^{77}Se NMR (76 MHz, $CDCl_3$) δ 6.8 (s); HRMS (ESI^+) calcd for $C_{47}H_{76}N_2O_{18}Se$ [M+Na] 1037.4337, found 1037. 4342.

The ^{77}Se chemical shifts in $CDCl_3$ of Si(Itome) and Se(IDEG) were 7.2 and 6.8 ppm (π-acceptor ability parameter, PAAP[9-11]), respectively, which is lower than found for the ligand IMes in the best SABRE catalyst in CD_3OD (31.6 ppm[11]) but relatively close to it when the full PAAP range is considered.[10]

SABRE of complex 1 in CD_3OD at 600 MHz

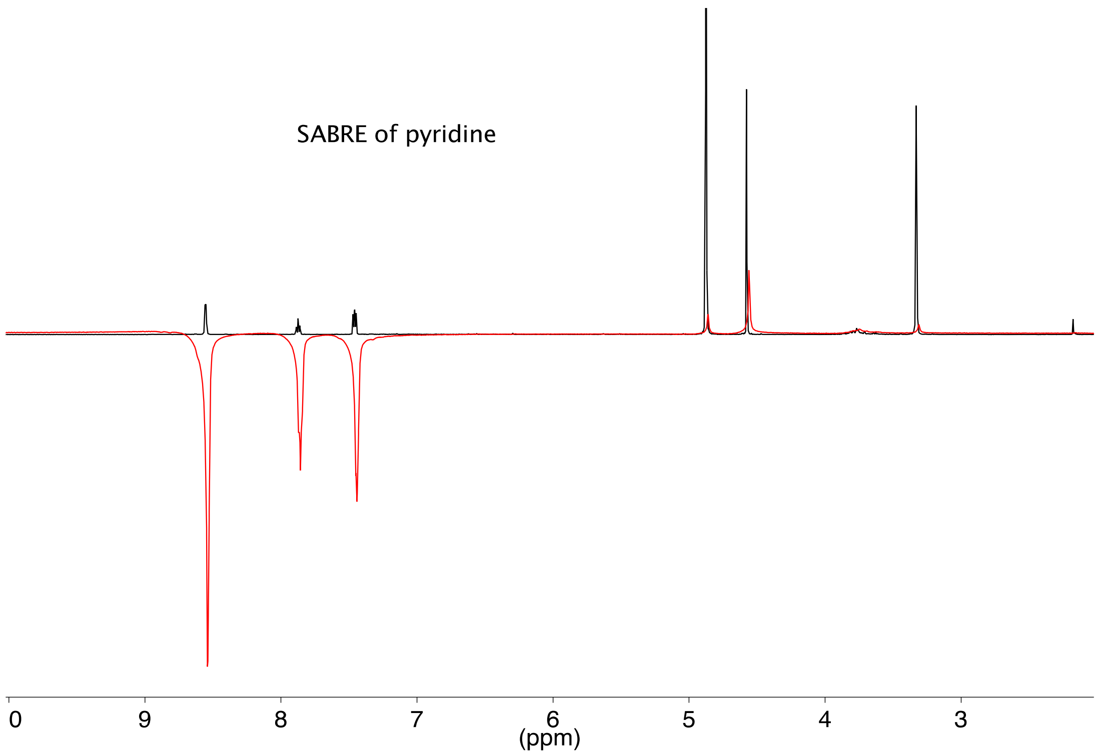

Figure S 10. ^{1}H NMR spectra on a 600 MHz spectrometer. Black trace: thermal spectrum of 1mM of **1** with 10mM of pyridine in CD_3OD. Red trace: polarized spectrum.

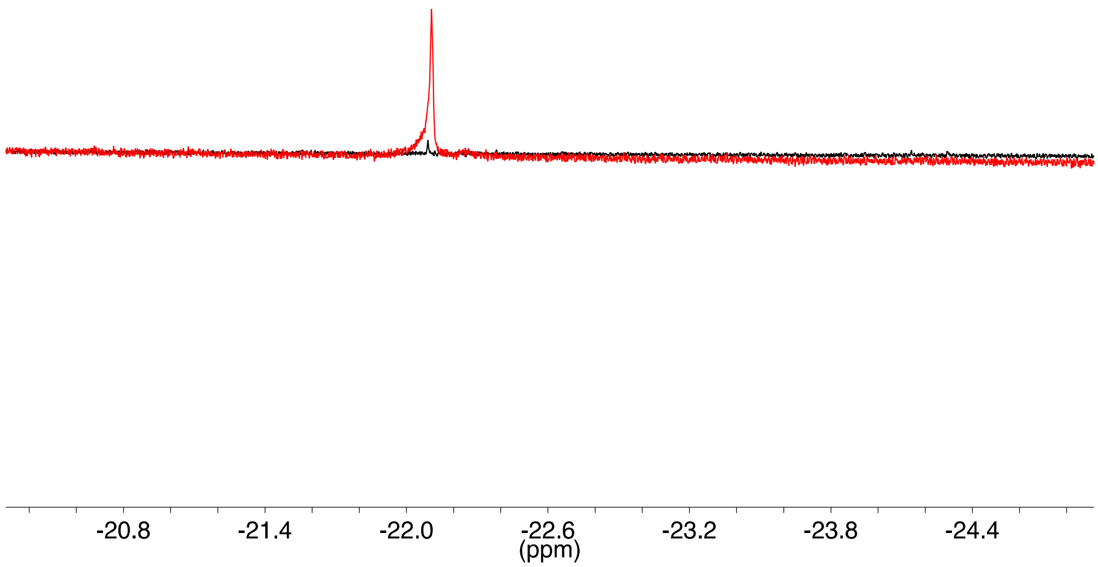

Figure S 11. ^{1}H SABRE experiments on a 600 MHz spectrometer at 65 G, hydride region, 1 mM **1**, 10 mM pyridine in CD_3OD, 5 bar of 50% parahydrogen, 48 h of pre-activation, manual shaking for 20 seconds. Black trace: thermal spectrum, red trace: polarized spectrum.

SABRE of complex 2 in D_2O at 300 MHz

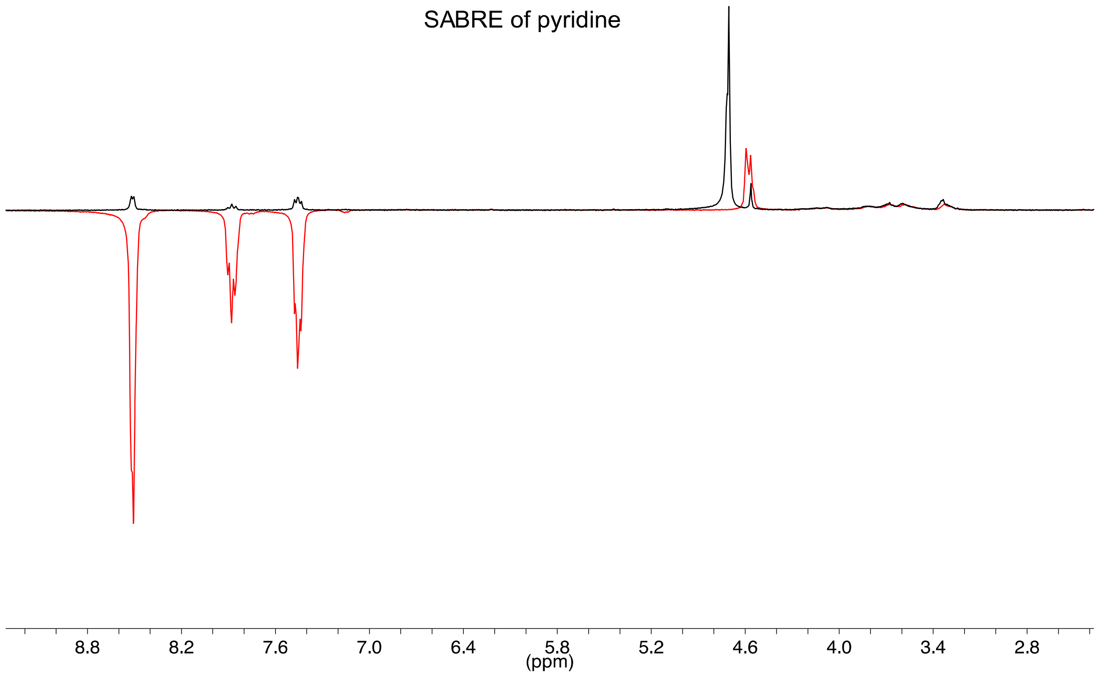

Figure S 12. ^{1}H NMR spectra on a 300 MHz spectrometer. Black trace: thermal spectrum of 1 mM of **2** with 10mM of pyridine in D_2O, red trace: polarized spectrum.

Only one hydride signal was typically seen after activation of **2**, with a chemical shift –22.17 in D_2O (see Figure S 13). The hydride signal remained persistent, indicating no H-D exchange.

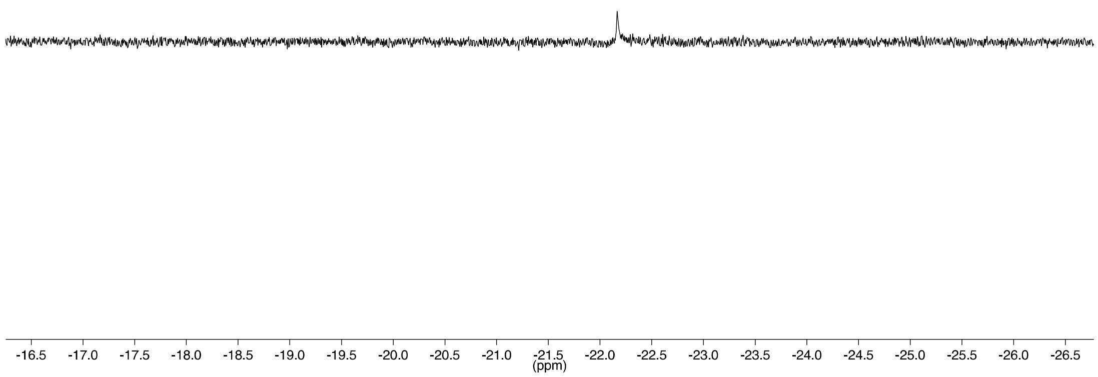

Figure S 13. ^{1}H NMR spectrum (hydride region) after the activation of 1mM of **2**, 10 mM of pyridine with 5 bar of parahydrogen in D_2O, measured at a 500 MHz spectrometer.

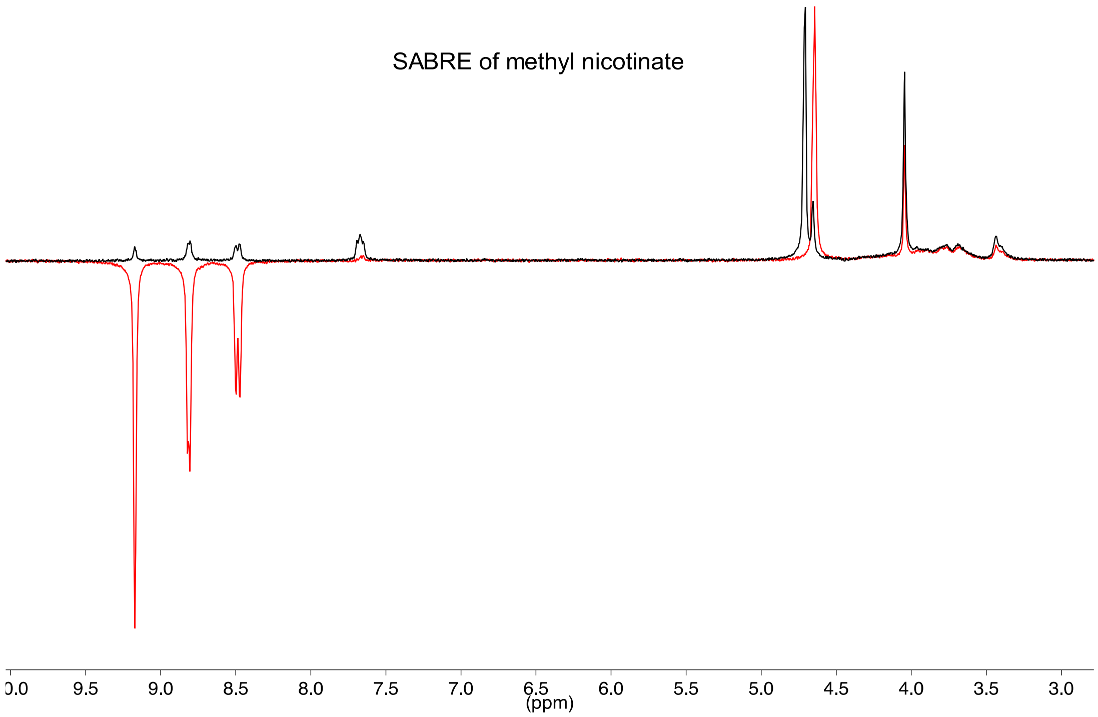

Figure S 14. ^{1}H NMR spectra on a 300 MHz spectrometer. Black trace: thermal spectrum of 0.66 mM of **2** with 13.32mM of methyl nicotinate in D_2O, red trace: polarized spectrum.

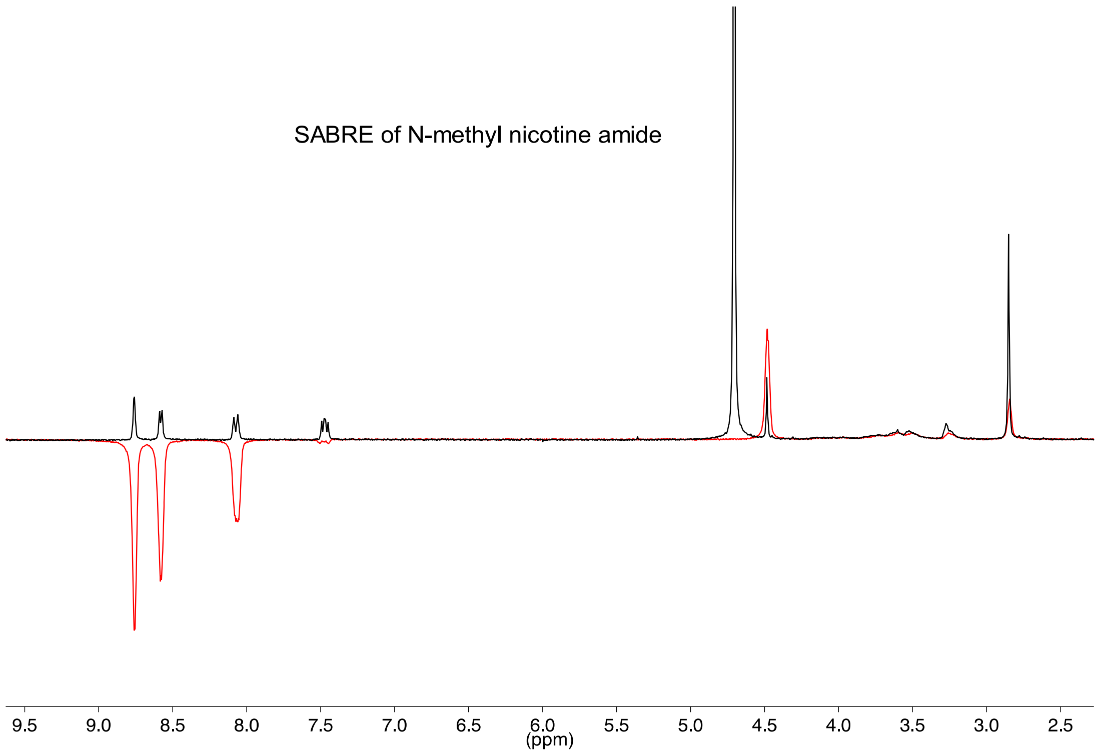

Figure S 15. ^{1}H NMR spectra on a 300 MHz spectrometer. Black trace: thermal spectrum of 0.66 mM of **2** with 13.32 mM of N-methyl nicotine amide in D_2O, red trace: polarized spectrum.

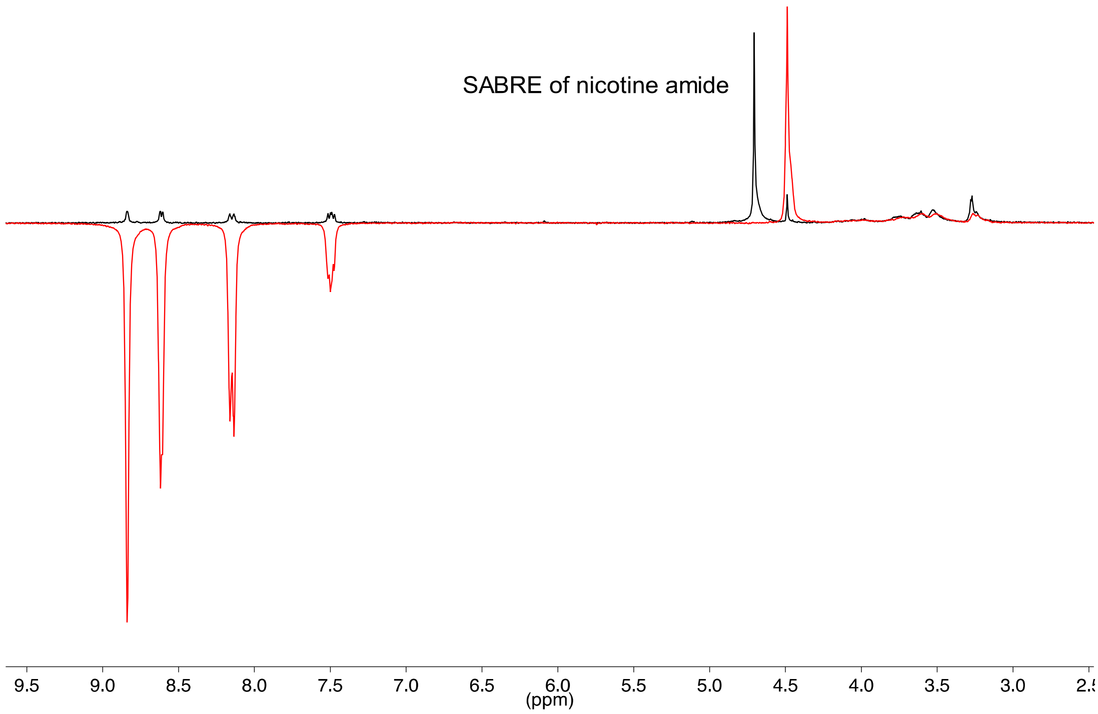

Figure S 16. ^{1}H NMR spectra on a 300 MHz spectrometer, 1 mM of **2** with 10 mM of N-methyl nicotine amide in D_2O after 24 h of activation, 5.5 bar of 92% *p*-H_2, heated to 60 °C for 30 seconds. Black trace: thermal spectrum, red trace: polarized spectrum.

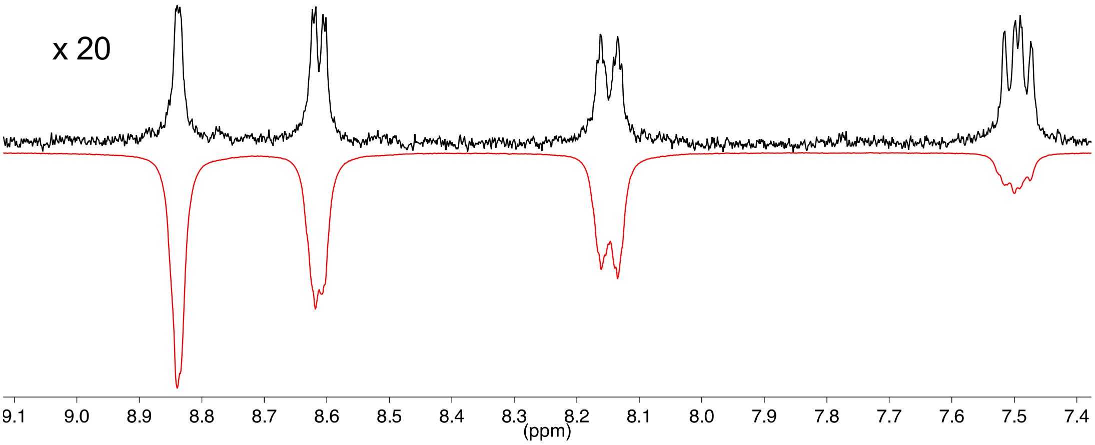

Figure S 17. SABRE of nicotine amide at 300 MHz, black trace: thermal signal enhanced 20-fold, red trace: polarized signal.

Uncategorized References

[1] K. F. H. Bonhoeffer, P, *Z. Phys. Chem. B* **1929**, *4*, 113.

[2] A. J. M. Duisenberg, L. M. J. Kroon-Batenburg, A. M. M. Schreurs, *J. Appl. Cryst.* **2003**, *36*, 220.

[3] G. M. Sheldrick, *SADABS. Program for Empirical Absorption Correction. University of Göttingen, Germany* **1996**.

[4] G. M. Sheldrick, *Acta Cryst.* **2008**, *A64*, 112-122.

[5] H. Ohta, T. Fujihara, Y. Tsuji, *Dalton Trans.* **2008**, 379-385.

[6] L. Xu, L. Shao, L. Chen, M. Hu, Y. Bi, *Chem. Lett.* **2010**, *39*, 1177-1179.

[7] B. J. A. van Weerdenburg, S. Glöggler, N. Eshuis, A. H. J. Engwerda, J. M. M. Smits, R. de Gelder, S. Appelt, S. S. Wymenga, M. Tessari, M. C. Feiters, B. Blümich, F. P. J. T. Rutjes, *Chem. Commun.* **2013**, *49*, 7388-7390.

[8] K. Harris Robin, D. Becker Edwin, M. Cabral de Menezes Sonia, R. Goodfellow, P. Granger, *Pure Appl. Chem.* **2001**, *73*, 1795-1818.

[9] A. Liske, K. Verlinden, H. Buhl, K. Schaper, C. Ganter, *Organometallics* **2013**, *32*, 5269-5272.

[10] S. V. C. Vummaleti, D. J. Nelson, A. Poater, A. Gomez-Suarez, D. B. Cordes, A. M. Z. Slawin, S. P. Nolan, L. Cavallo, *Chem. Sci.* **2015**, *6*, 1895-1904.

[11] B. J. A. van Weerdenburg, N. Eshuis, M. Tessari, F. P. J. T. Rutjes, M. C. Feiters, *Dalton Trans.* **2015**, *44*, 15387-15390.
